# Evaluating Large Language Model–Based Automated Scoring in a Voice-Based Virtual Standardized Patient Platform for Medical Students: Cross-Sectional Agreement Study

**DOI:** 10.2196/95578

**Published:** 2026-09-24

**Authors:** Xiaoxing Gao, Xiaoming Huang, Rongrong Hu, Li Zhang, Huiting Liu, Bingqing Zhang, Chong Wei, Wei Qiu, Mengyu Zhang, Xuefeng Sun

**Affiliations:** 1 Department of Pulmonary and Critical Care Medicine, Peking Union Medical College Hospital, 1 Shuaifuyuan, Dongcheng District, Beijing, 100730, China, 86 10 6915 5000; 2 Department of General Internal Medicine, Peking Union Medical College Hospital, Beijing, China; 3 Department of Nephrology, Peking Union Medical College Hospital, Beijing, China; 4 Department of Rheumatology, Peking Union Medical College Hospital, Beijing, China; 5 Department of Infectious Disease, Peking Union Medical College Hospital, Beijing, China; 6 Department of Haematology, Peking Union Medical College Hospital, Beijing, China; 7 Department of Medical Oncology, Peking Union Medical College Hospital, Beijing, China; 8 Department of Neurology, Peking Union Medical College Hospital, Beijing, China; 9 Department of Internal Medicine, Peking Union Medical College Hospital, Beijing, China

**Keywords:** large language model, virtual standardized patient, information gathering, communication skills, rater variability, mixed-effects model

## Abstract

**Background:**

Large language model (LLM)–powered virtual standardized patients (VSPs) enable scalable clinical skills practice, but the validity of AI-generated scores relative to faculty ratings remains unclear.

**Objective:**

This study aimed to assess agreement between LLM-generated and faculty ratings of history-taking and communication performance and to examine the influence of rater and case heterogeneity.

**Methods:**

In this cross-sectional study, 92 fourth-year medical students completed one of three 15-minute voice-based VSP cases (fever, diarrhea, and cough). Ten blinded faculty raters scored performance (0‐100 points total; 0‐50 points per domain). AI scores were generated by DeepSeek-V3 using a calibrated prompt. Agreement was evaluated using mixed-effects models, intraclass correlation coefficients (ICC [2,1]), Spearman correlations, mean absolute error (MAE), Bland-Altman analysis, and variance partition coefficients (VPC).

**Results:**

Median total scores were similar for AI and faculty (median 93.0, IQR 89.0-95.0 vs median 94.0, IQR 91.0-95.0). Rater variability accounted for 37% of residual variance in faculty total scores (VPC=0.37). AI total scores were positively associated with faculty total scores (β=0.37, 95% CI 0.26‐0.48; *P*<.001; Spearman ρ=0.50, 95% CI 0.34‐0.65). Absolute agreement was moderate (ICC[2,1]=0.51, 95% CI 0.34‐0.65), with MAE of 3.11 points. Mixed-effects Bland-Altman analysis showed a small, not statistically significant mean bias (1.26 points, 95% CI −0.48 to 3.01; *P*=.16) and 95% limits of agreement from −4.95 to 7.48 (width=12.43 points), with proportional bias (β_proportional bias=−0.55; *P*<.001). Agreement was stronger for information gathering (β=0.46; ρ=0.49; ICC=0.54; VPC=0.23) than for communication (β=0.27; ρ=0.28; ICC=0.29; VPC=0.52). A sensitivity analysis in the lowest quartile showed attenuated but consistent agreement (ICC=0.38).

**Conclusions:**

LLM-based scoring in a VSP showed moderate agreement with faculty ratings, performing better for information gathering than for communication. Due to rater and case heterogeneity, ceiling effects, and proportional bias, this method is suitable for formative use and enhanced sampling in programmatic assessment but not for independent, high-stakes summative decisions.

## Introduction

### Background

History-taking and physician-patient communication are core competencies in undergraduate medical education. Traditional teaching relies on faculty-facilitated role-play and standardized patients (SPs), which are resource-intensive and constrained by faculty time and SP availability. As a result, students often have limited opportunities for repeated practice and timely feedback.

Virtual standardized patients (VSPs) are computer-based simulations designed to portray patients with defined clinical presentations and respond dynamically to learner inquiries. They offer a scalable supplement or alternative. Virtual patients have long been used to support history-taking and clinical reasoning, and reviews show positive learner perceptions and modest gains in knowledge and skills compared with traditional methods [1-3]. Earlier systems largely used branching logic and rule-based scoring. Recent large language models (LLMs) can support free-text, contextually appropriate dialogue, enabling more natural and flexible patient simulations [4].

Beyond dialogue, LLMs can generate automated scores and feedback, potentially increasing feedback frequency while reducing faculty workload [5]. However, the usefulness of automated assessment depends on validity and reliability. For summative uses (eg, progression decisions), strong agreement with expert judgment and robust validity evidence are essential; for formative use, moderate agreement may be acceptable if feedback is timely, specific, and actionable [6].

Notably, even trained human raters in objective structured clinical examinations (OSCEs) and workplace-based assessments often show only moderate interrater reliability and substantial variability in severity and leniency [7,8]. Therefore, AI-human agreement should be interpreted in the context of human-human variability, rather than assuming a flawless human “gold standard.” Few empirical studies have systematically compared LLM-driven scoring of clinical communication with human examiners in authentic teaching settings—especially within integrated VSP platforms [5,9,10].

We developed an LLM-based VSP system for voice-based history-taking and communication practice. Students conducted spoken interviews with an LLM-driven virtual patient; the system then generated scores and narrative feedback. We evaluated the system by comparing LLM-generated scores with faculty ratings across multiple instructional groups and clinical cases.

This study aimed to evaluate the agreement between LLM-generated and faculty ratings of history-taking and communication performance in an LLM-powered VSP platform and to examine how rater and case heterogeneity influenced agreement to determine appropriate educational use boundaries for LLM-based scoring.

### Research Questions

This study addressed three questions: (1) How closely do LLM total scores agree with faculty ratings? (2) How do rater and case factors affect AI-human agreement? (3) Is the psychometric agreement between LLM-based scoring and faculty ratings sufficient to support formative and/or summative assessment uses?

## Methods

### Study Design and Setting

This cross-sectional observational study was conducted at Peking Union Medical College Hospital. Each student completed a single VSP encounter via a voice-based dialogue. In each encounter, the VSP acted as the patient, while students role-played the physician to conduct a history-taking interview. Encounters were limited to 15 minutes. Immediately after the encounter, the supervising faculty member provided ratings while blinded to AI-generated scores.

### Participants

Ninety-two fourth-year medical students enrolled in a diagnostics course participated. Prior to the experiment, all participants had completed 7 weeks of history-taking training, including 1 human SP practice session per week, and they were offered voluntary access to an LLM-powered VSP platform aligned with the SP curriculum. Students could initiate unlimited, untimed multiturn interviews with any case, at any time, outside scheduled SP sessions.

As preparation for the end-of-term history-taking examination, we conducted this VSP-based exercise. Students were informed that this was an AI-simulated practice session; they were not told that AI scores would be compared with faculty ratings for research. Ten faculty raters scored students using a standardized rubric, with brief orientation but no formal calibration.

### LLM-Powered VSP System

The VSP system used DeepSeek-V3 (DeepSeek AI, accessed via API, temperature=0.0) for scoring, Doubao-1.5 (ByteDance) for patient dialogue, paraformer-realtime-v2 (Alibaba) for automatic speech recognition (ASR), and CosyVoice-v2 (Alibaba) for text-to-speech synthesis. Prior to deployment, we iteratively calibrated the LLM scoring prompt using 45 historical records from the 2024 academic year (independent cohort, no overlap in students, cases, or raters). The five-stage calibration involved (1) rubric-aligned prompt drafting, (2) initial testing on 15 records, (3) item-level discrepancy analysis, (4) iterative refinement targeting systematic errors, and (5) validation on the held-out 30 records. The prompt was frozen before deployment. The complete prompt and detailed calibration procedure are provided in Multimedia Appendix 1.

Three clinical cases (fever, diarrhea, and cough) were developed by the same clinical education team to represent common outpatient presentations of comparable complexity and expected duration (15 min). Participants were randomly assigned to one of 3 cases using a simple randomization procedure (drawing identical paper lots). Group sizes varied (case 1: n=32; case 2: n=35; case 3: n=25) because of random allocation.

### Scoring Dimensions and Rubrics

Immediately after each encounter, 2 independent 100-point scores were generated using the same 50:50 weighting for information gathering and communication. Information gathering (0‐50 points) assessed completeness and relevance of the clinical history through approximately 40 checklist items. Communication (0‐50 points) assessed process quality through 10 criteria, including clinical reasoning, question appropriateness, summarization, transitional language, and humanistic care. Communication criteria were weighted from 3 to 8 points based on clinical importance; the complete rubric is provided in Multimedia Appendix 1.

### Statistical Analysis

Analyses were conducted in R (version 4.3.2; R Foundation for Statistical Computing). The unit of analysis was the student (n=92); each student contributed 1 paired set of AI-generated and faculty-assessed scores from a single VSP encounter. Because each encounter was scored by 1 faculty rater, clustering by rater was handled using mixed-effects models with rater-level random intercepts. Given bounded (0‐100) scores with ceiling tendencies, results were summarized using median (25th-75th percentile). All tests were 2-sided (α=.05).

The primary analysis used a linear mixed-effects model: human_total ~ AI_total + case + (1|rater). A random-slope specification (AI_total|rater) was explored; singular fits were interpreted as insufficient information to support between-rater slope variability. Between-rater clustering was summarized using the variance partition coefficient (VPC). Absolute agreement was summarized with intraclass correlation coefficient (ICC[2,1]; 2-way random-effects, single-measure, absolute agreement). A mixed-effects Bland-Altman model estimated mean bias and 95% limits of agreement, with tests for proportional bias. Full model specifications are in Multimedia Appendix 2.

All analyses were repeated for information gathering and communication subscales. Prespecified sensitivity analyses refit the primary model using robust mixed-effects estimation and refit without case adjustment. Additional summaries included Spearman ρ and mean absolute error (MAE).

### Student Feedback Survey

After completing the VSP encounter, students were invited to complete an anonymous online feedback questionnaire. The survey assessed 4 dimensions: overall satisfaction (0‐10 scale), perceived convenience of the VSP platform (1‐5 Likert scale), perceived realism compared with human SP interactions (1‐5 Likert scale), and perceived feedback value relative to human SPs (1‐5 Likert scale). Responses were collected via a web-based form (Wenjuanxing) immediately after each encounter. Descriptive statistics (mean and response distribution) were computed for each item. The questionnaire was developed de novo for this study to capture platform-specific user experience and was not adapted from a previously validated instrument.

### Ethical Considerations

This study used deidentified student data from routine teaching activities. The Peking Union Medical College Hospital Ethics Committee approved this secondary analysis (I-26PJ0511) and granted a waiver of written informed consent. Students had been informed that their encounters might be used for educational research. The study adhered to the principles of the Declaration of Helsinki.

## Results

### Descriptive Statistics

**Figure 1.**
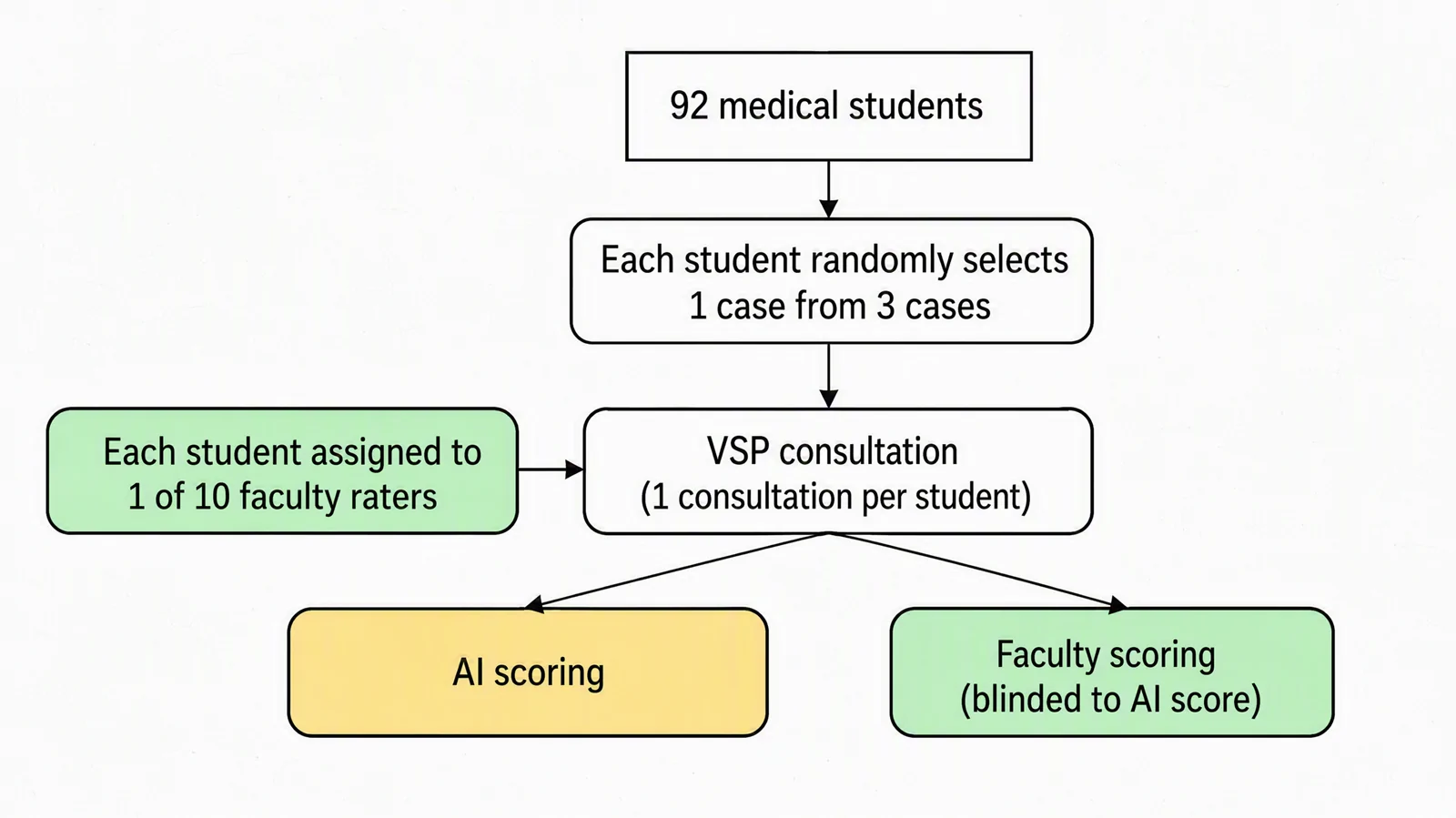
Flowchart of the study design. VSP: virtual standardized patient.

The study design flowchart is shown in Figure 1. All 92 students successfully completed their VSP encounters without system crashes or encounter restarts. No encounters were excluded due to technical failures. Occasional ASR errors were informally noted by supervising faculty, primarily affecting medical terminology and rapid speech; students had been trained to correct such errors by repeating or rephrasing their questions.

Ninety-two medical students each contributed 1 paired observation and were rated by 1 of 10 independent raters (8‐10 students each, median 9, IQR 9-10). Students were randomized to 3 cases (case 1: n=32; case 2: n=35; case 3: n=25). Overall scores were high, with evidence of ceiling effects: median AI total score 93.0 (IQR 89.0-95.0), median human total score 94.0 (IQR 91.0-95.0); 17 (18.5%) students scored above 95 on AI totals and 17 (18.5%) on human totals, with 6 (6.5%) students scoring above 95 on both. AI total scores ranged from 77 to 100 and human total scores from 84 to 99. Score distributions were compressed (total-score SDs 3.4-4.6: AI 4.6, human 3.4; subscale SDs 1.5-3.0: AI information 3.0, human information 2.9, AI communication 3.0, human communication 1.5). Median total scores were similar across cases, whereas rater medians varied substantially (AI 89.0‐94.0; human 87.2‐95.0), indicating meaningful between-rater severity differences (Table 1). Histograms are provided in Multimedia Appendix 3.

**Table 1.** Descriptive statistics of AI and human ratings across competency dimensions.

Group	AI total score	Human total score	AI information score	Human information score	AI communication score	Human communication score
Overall (n=92), median (IQR)	93.0 (89.0-95.0)	94.0 (91.0-95.0)	46.0 (44.0‐48.0)	46.0 (44.5‐47.0)	47.0 (45.0‐49.0)	48.0 (47.0‐49.0)
Case, median (IQR)						
Case 1 (n=32)	92.0 (89.8-95.2)	94.0 (91.5‐95.2)	47.0 (45.0‐48.0)	46.5 (44.9‐47.2)	46.5 (44.8‐48.2)	48.0 (47.0‐49.0)
Case 2 (n=35)	93.0 (89.5‐95.0)	94.0 (92.0‐95.0)	46.0 (44.0‐47.0)	46.0 (45.0‐46.2)	47.0 (46.0-49.0)	48.0 (46.0‐49.0)
Case 3 (n=25)	92.0 (89.0‐94.0)	93.0 (88.5‐95.0)	45.0 (44.0‐47.0)	45.0 (40.5‐47.0)	47.0 (44.0‐48.0)	48.0 (47.0‐49.0)
Rater, median (IQR)						
Rater 1 (n=9)	94.0 (91.0‐95.0)	95.0 (94.0‐95.0)	47.0 (45.0‐47.0)	46.0 (46.0‐47.0)	49.0 (46.0‐49.0)	48.0 (48.0‐49.0)
Rater 2 (n=9)	94.0 (92.0‐96.0)	94.0 (93.0‐94.0)	48.0 (47.0‐49.0)	46.0 (44.5‐47.0)	46.0 (45.0‐47.0)	48.0 (47.0‐48.0)
Rater 3 (n=9)	93.0 (91.0‐95.0)	95.0 (95.0-97.0)	48.0 (46.0‐49.0)	47.0 (46.0‐47.0)	47.0 (44.0‐47.0)	49.0 (49.0‐49.0)
Rater 4 (n=9)	93.0 (90.0‐95.0)	91.0 (90.0‐93.5)	45.0 (44.0‐47.0)	45.0 (44.5‐45.0)	48.0 (44.0‐50.0)	48.0 (46.0‐49.0)
Rater 5 (n=8)	93.0 (89.0‐94.0)	94.5 (92.6‐96.5)	47.0 (45.0‐48.0)	45.8 (44.0‐47.8)	46.0 (43.5‐46.1)	48.8 (48.0‐49.0)
Rater 6 (n=10)	93.0 (90.5‐95.0)	94.0 (93.0‐94.8)	47.0 (45.2‐47.0)	47.0 (47.0‐48.0)	47.0 (46.2‐48.0)	47.0 (46.0‐47.0)
Rater 7 (n=10)	91.0 (89.2‐93.5)	87.2 (86.2‐91.2)	44.0 (41.2‐45.0)	40.5 (39.0‐44.5)	47.5 (45.5‐49.8)	47.0 (46.0‐47.9)
Rater 8 (n=10)	92.5 (88.2‐94.8)	91.2 (90.2‐92.0)	45.0 (43.2‐45.8)	44.8 (44.0‐45.8)	48.5 (47.2‐49.0)	46.5 (46.0‐47.8)
Rater 9 (n=8)	93.0 (92.0‐95.2)	95.0 (93.5‐96.2)	47.0 (46.0‐47.0)	47.0 (46.8‐48.0)	47.0 (45.8‐48.2)	48.0 (46.0‐49.0)
Rater 10 (n=10)	89.0 (86.5‐91.2)	92.0 (89.2‐95.8)	44.0 (42.2‐45.0)	44.0 (40.2‐45.8)	44.5 (43.2‐47.8)	49.0 (49.0‐49.8)

### Rater Clustering

A linear mixed-effects model with fixed effects for the AI total score and case and a random intercept for rater showed notable rater dependence (Var[rater]=3.05; Var[residual]=5.29), yielding a VPC of 0.37. This indicates that 37% of the unexplained variance in human total scores was attributable to between-rater differences in average scoring severity or leniency. Accounting for rater clustering improved fit versus ordinary least squares (ΔAkaike information criterion=19.10), supporting the presence of nontrivial rater heterogeneity [11].

### Agreement Between AI and Human Total Scores

**Figure 2.**
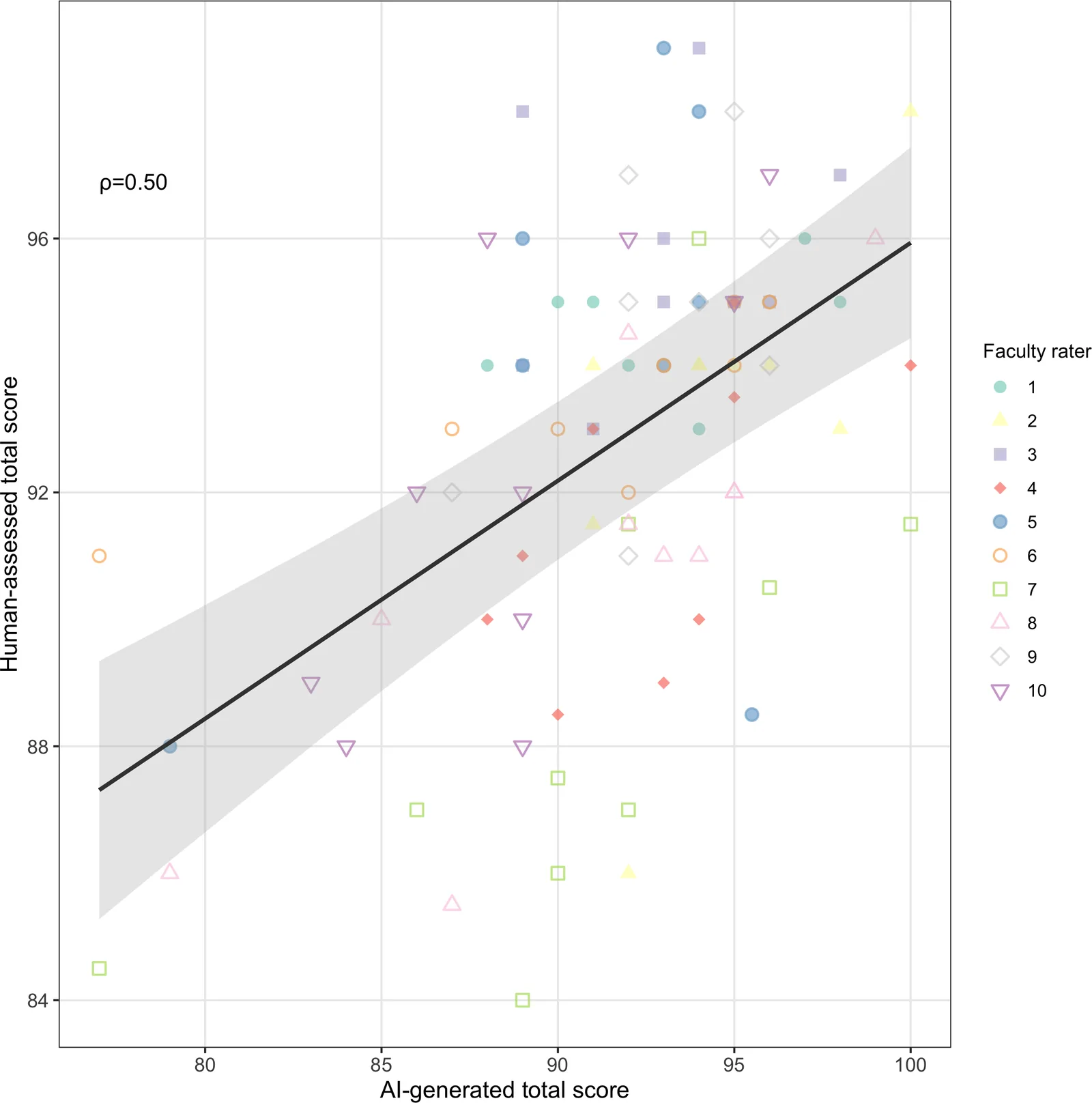
Scatter plot showing the association between AI-generated total scores and human-assessed total scores. Each point represents an individual medical student’s score pair (n=92). The solid black line indicates the linear regression fit from the mixed-effects model (β=0.37, 95% CI 0.26‐0.48; *P*<.001), and the gray shaded area represents the 95% CI for the regression line.

**Figure 3.**
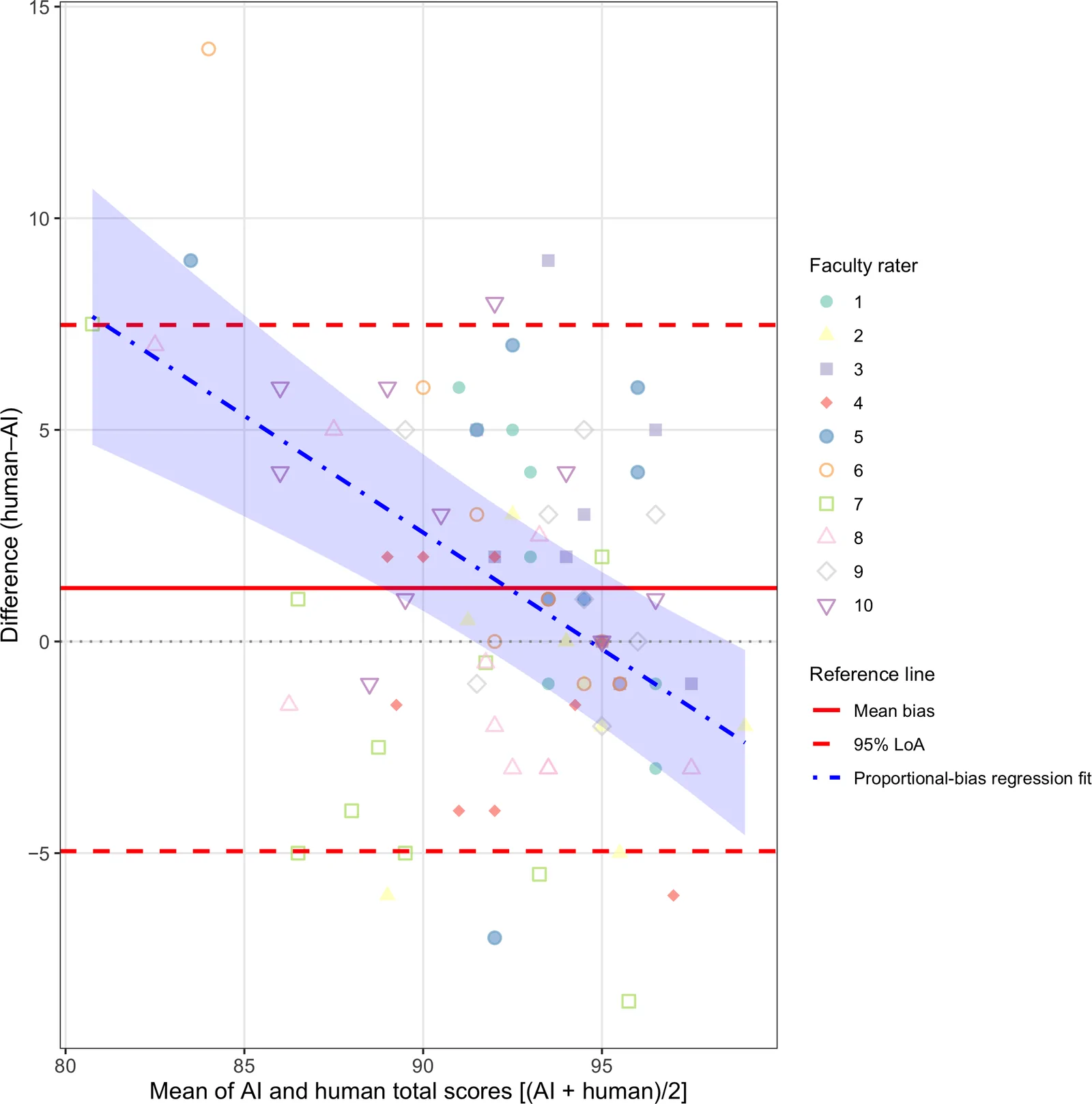
Mixed-effects Bland-Altman plot for AI and human total scores. The solid red line indicates the mean bias (1.26 points, 95% CI −0.48 to 3.01), which did not reach statistical significance at the group level (*P*=.16). The dashed red lines represent the 95% limits of agreement (LoA; −4.95 to 7.48; width=12.43 points). The blue line shows proportional bias (β_proportional bias=−0.55, 95% CI −0.75 to −0.35; *P*<.001), indicating that AI assigned relatively higher scores at the lower end of the performance distribution.

In the primary mixed model, the AI total score was positively associated with the human total score (β=0.37, 95% CI 0.26‐0.48; *P*<.001; Table 2 and Figure 2). Case indicators were not statistically significant (Case 2: *P*=.86; Case 3: *P*=.23). Agreement was moderate: Spearman ρ=0.50, ICC(2,1)=0.51, and MAE=3.11 points. Mixed-effects Bland-Altman analysis showed a mean bias that did not reach statistical significance (1.26 points, 95% CI −0.48 to 3.01; *P*=.16) but wide 95% limits of agreement (−4.95 to 7.48; width=12.43 points), indicating educationally meaningful individual-level discrepancies. Proportional bias was present (β_proportional bias=−0.55; *P*<.001), with AI assigning relatively higher scores at the lower end of the performance distribution (Figure 3).

**Table 2.** Model-based association and AI-human agreement.

Outcome	LMM^a^, β (95% CI)	*P* value	Spearman ρ	ICC^b^(2,1)	Mean bias (human–AI)	Conditional 95% LoA^c^	Proportional bias, β_proportional bias	Proportional bias, *P* value
Total score	0.37 (0.26‐0.48)	<.001	0.50	0.51	1.26	−4.95 to 7.48	−0.55	<.001
Information	0.46 (0.30‐0.63)	<.001	0.49	0.54	−0.38	−5.52 to 4.77	−0.10	.40
Communication	0.27 (0.19‐0.34)	<.001	0.28	0.29	1.29	−1.69 to 4.28	−0.94	<.001

a LMM: linear mixed-effects model.

b ICC: intraclass correlation coefficient.

c LoA: limits of agreement.

### Subgroup Patterns

**Figure 4.**
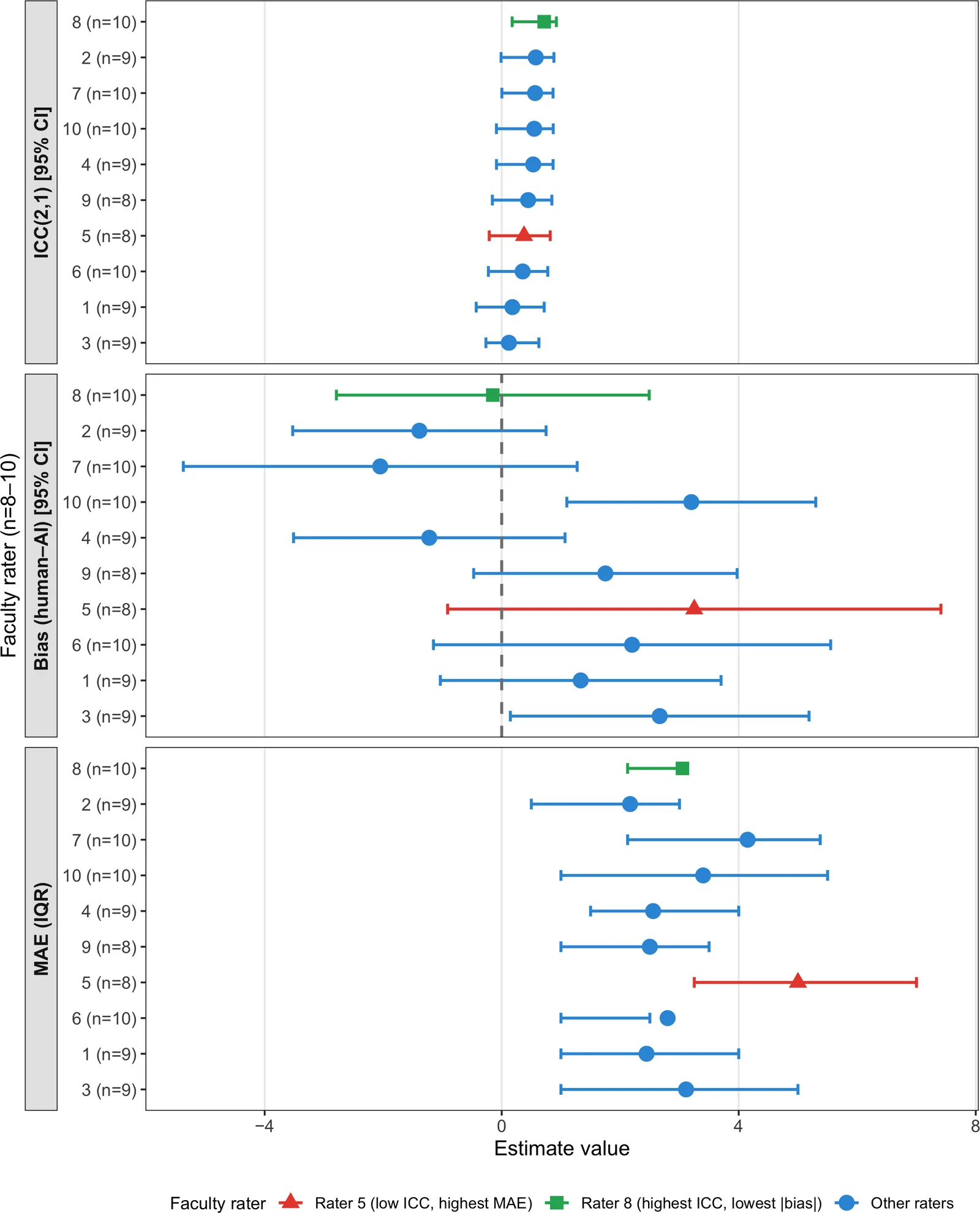
Forest (dot-whisker) plot of rater-level heterogeneity in AI-human agreement for total scores. Each row represents an independent rater (n=8‐10), ordered by ICC(2,1) point estimate. Points show estimates and whiskers show 95% CIs for ICC(2,1), bias (mean Human–AI; vertical line at 0), and mean absolute error (MAE). Given small per-rater sample sizes, results are descriptive. ICC: intraclass correlation coefficient.

Rater-stratified agreement varied widely (ICC[2,1] estimates 0.57‐0.93; Spearman ρ 0.21‐0.93; MAE 2.17‐5.00) with imprecise estimates (n=8‐10 per rater). Mean AI-human bias also varied in direction across raters (–2.05 to 3.25), consistent with rater-specific scoring stringency or leniency or local calibration effects (Figure 4).

Case-stratified results suggested highest agreement in case 1 (ICC=0.63; ρ=0.62; MAE=2.83) and lowest in case 3 (ICC=0.36; ρ=0.32; MAE=4.24). These case-level estimates should be interpreted descriptively given limited per-subgroup sample sizes.

### Sensitivity Analyses

Prespecified sensitivity analyses examined model robustness. Refitting the primary mixed-effects model with robust Huber estimation yielded a nearly identical AI coefficient (β=0.35, 95% CI 0.24‐0.46; *P*<.001). Omitting case adjustment also produced an essentially unchanged estimate (β=0.36, 95% CI 0.25‐0.47; *P*<.001). Full details are provided in Multimedia Appendix 4.

### Subscale Analyses

AI-human agreement differed by subscale, with stronger alignment for information gathering than for communication (Table 2). For information gathering, β=0.46 (*P*<.001); ρ=0.49; ICC=0.54 (95% CI 0.38‐0.67); VPC=0.23. For communication, β=0.27 (*P*<.001); ρ=0.28; ICC=0.29 (95% CI 0.08‐0.47); VPC=0.52, indicating greater rater dependence and lower reproducibility.

### Student Feedback

Of the 92 participating students, 63 (68.5%) completed the postencounter feedback questionnaire. Overall satisfaction was moderate (mean 6.4, SD 1.9 on a 10-point scale). Students highly valued the convenience of the VSP platform (mean 4.1, SD 0.8 on a 5-point scale). However, perceived realism was low (mean 1.8, SD 0.9 on a 5-point scale), and perceived feedback value relative to human SPs was below the scale midpoint (mean 2.4, SD 1.1 on a 5-point scale). Full survey results, including item-level response distributions, are provided in Multimedia Appendix 3.

## Discussion

### Principal Findings

In this study of a voice-based, LLM-powered VSP used by fourth-year medical students, 3 main findings emerged. First, AI-generated and faculty-assigned total scores showed moderate agreement, with stronger alignment for information gathering than for communication. Second, rater differences accounted for 37% of residual variance in total scores and 52% in communication scores, indicating that apparent AI-human disagreement partly reflects human-human variability. Third, proportional bias indicated that AI tended to assign relatively higher scores at the lower end of the performance distribution.

### Interpretation and Psychometric Implications

From a psychometric perspective, the magnitude of AI-human agreement observed here does not support unsupervised, high-stakes use for individual learners. Although mean bias was small and did not reach statistical significance (1.26 points, 95% CI −0.48 to 3.01; *P*=.16), and the 95% limits of agreement spanned 12.43 points on a 100-point scale, wide enough that an individual learner could plausibly be classified differently depending on whether the AI or a faculty rater provided the score. Ceiling effects further complicate interpretation: with 18% of students scoring above 95 and total score SDs of only 3.4 to 4.6 points, the observed ICC of 0.51 should be treated as a lower-bound estimate. A range-restriction sensitivity analysis (Thorndike Case 2 correction) yielded a corrected ICC of approximately 0.72 (Multimedia Appendix 2). Proportional bias (AI assigning relatively higher scores to lower-performing students) further cautions against high-stakes use, particularly for communication skills.

At the same time, the overall direction and robustness of the AI-human association, which were stronger than those often observed between individual human raters in clinical performance assessments, suggested practical utility for low-stakes formative contexts [5,6,12,13]. In these contexts, the primary goal is not precise interchangeability with a single rater but scalable feedback and increased sampling of performance.

### Validity Argument

This study provided criterion-related validity evidence by quantifying associations with faculty judgments. However, it addressed psychometric feasibility (score agreement) rather than educational feasibility (impact on learning outcomes), which requires dedicated longitudinal studies. Validity also depends on content alignment, response process coherence, and consequences [6]; these domains require further investigation before broader adoption—especially for higher-stakes purposes—can be justified.

### Subscale-Specific Performance and Equity

Information gathering showed higher AI-human agreement and lower rater variance than communication. This aligned with the relative objectivity of checklist-based content compared with the judgment-laden nature of communication behaviors [14]. In practice, this makes AI scoring potentially useful for identifying omitted history elements, quantifying completeness, and tracking progress over time.

Communication scoring, however, remains challenging. Subjective judgments about rapport, empathy, and professionalism depend on nuanced, context-sensitive interpretation that current LLMs may not reliably replicate [15,16]. This is also an equity-relevant domain: communication judgments—whether by humans or AI—may be sensitive to accent, language proficiency, culturally patterned interaction styles, or disability-related differences. These potential differential effects should be tested explicitly rather than assumed away [17].

### Rater Heterogeneity and Faculty Development

Rater-stratified results showed wide variability in AI-human agreement across faculty, reinforcing that human raters themselves are not interchangeable. Because students were nested within raters in this design, a single human score cannot be treated as an error-free reference; rather, it reflects one draw from a distribution of possible faculty judgments. This variability also limited generalizability: AI-human agreement observed here depended on the particular mix of raters and their scoring tendencies [18,19].

Rather than treating faculty ratings as a fixed “gold standard,” a more defensible target is whether AI scores fall within the range of acceptable human variation. If AI-human disagreement is comparable to human-human disagreement, AI scores can be considered psychometrically equivalent to an additional human rater—a standard that has been proposed for automated scoring in other domains [20-22].

### Relation to Prior Work

Prior work on LLMs in medical education has emphasized written examination performance [23-25]. This study instead evaluated an LLM as an assessor of interactive clinical performance, foregrounding questions of rater variability and validity for performance scores. Our results aligned with emerging evidence that AI-human agreement in clinical communication assessment is moderate rather than excellent [9,10,26], and they extended that literature by quantifying how much apparent AI-human disagreement reflected human-human variability [6]. Importantly, moderate AI-human agreement does not necessarily imply invalid AI scoring; rather, it highlights that both AI and human raters bring distinct sources of error and bias [20-22].

### Limitations

This study has several limitations. Data were drawn from a single institution with predominantly high-performing students, and ceiling effects may limit generalizability to other learner populations. Each student completed only 1 encounter evaluated by a single faculty rater, precluding assessment of within-student stability and human interrater reliability. Faculty received brief rubric orientation without structured calibration, which may have introduced variability in scoring standards. Although we examined quantitative score agreement, we did not evaluate the quality or educational impact of AI-generated narrative feedback. Students’ awareness of interacting with an AI system may have influenced communication behaviors. ASR error rates were not systematically recorded, limiting assessment of potential transcription bias. The scoring prompt was aligned with local faculty norms, and the analyses relied on specific LLM models, prompts, and cases; these factors may limit generalizability across institutions and over time, underscoring the need for multisite validation. Finally, the student feedback questionnaire was newly developed for this study and has not undergone formal psychometric validation (eg, content validity or reliability testing); the survey results should therefore be interpreted as exploratory and descriptive.

### Student Experience and Perceived Utility

Student feedback on the VSP platform highlighted a tension between convenience and authenticity. While learners appreciated the on-demand accessibility of AI-based practice, they rated the realism of AI-simulated patient interactions and the perceived value of AI-generated feedback lower than comparable ratings for human SP encounters. These perceptions likely reflect current limitations in conversational naturalness, which may attenuate engagement and learning transfer. Improving dialogue fidelity and closing the perceived feedback quality gap represent key priorities for VSP refinement. The juxtaposition of moderate satisfaction with low perceived realism suggests that learner experience is shaped by factors beyond scoring accuracy alone, underscoring the need for multidimensional evaluation of VSP systems.

### Practical Implications

Given the current evidence, we suggest that LLM-based scoring in VSPs can be used to prioritize practice opportunities and deliver rapid feedback—especially for information gathering—where scalability and frequent practice are central and where perfect interchangeability with a faculty score is not required. High-stakes use should remain human-supervised and, if used at all, should be embedded in hybrid workflows where AI provides an additional perspective rather than a final decision.

Within programmatic assessment, AI scores may be most defensible for increasing sampling density, flagging learners for coaching, and triangulating with other evidence (OSCEs, workplace-based assessments, and supervisor narratives), rather than serving as a standalone progression metric.

### Future Directions

Future work should develop behavioral benchmarks that disentangle AI error from legitimate variation in faculty judgment, characterize differential item functioning across student subgroups, and test rater calibration interventions that leverage AI scores to improve human rating consistency.

### Conclusions

In this undergraduate medical course, an LLM-based VSP generated history-taking and communication scores that showed moderate agreement with human raters, with greater convergence for information gathering than for communication skills. Substantial variability among human raters indicated that human scoring was itself an imperfect reference standard. Overall, these findings supported LLM-based scoring as a feasible, scalable tool for formative assessment and practice, while cautioning against unsupervised high-stakes application. Ongoing validation should prioritize rater calibration, communication scoring refinement, and equity auditing as these technologies mature in medical education.

## Supplementary material

10.2196/95578 Multimedia Appendix 1 Complete large language model scoring prompt and iterative calibration procedure.

10.2196/95578 Multimedia Appendix 2 Detailed statistical model specifications.

10.2196/95578 Multimedia Appendix 3 Score distribution histograms and student feedback survey results.

10.2196/95578 Multimedia Appendix 4 Rater-stratified agreement metrics, sensitivity analyses, and lower-quartile analysis.

## Abbreviations

ASR: automatic speech recognition
ICC: intraclass correlation coefficient
LLM: large language model
MAE: mean absolute error
OSCE: objective structured clinical examination
SP: standardized patient
VPC: variance partition coefficient
VSP: virtual standardized patient
