# Supplementary material for: Evaluating Large Language Model–Based Automated Scoring in a Voice-Based Virtual Standardized Patient Platform for Medical Students: Cross-Sectional Agreement Study
Source: JMIR Med Educ. 2026 Sep 24;12:e95578. doi: 10.2196/95578 (PMC13606323; doi:10.2196/95578)
Supplement: Supplementary file 1 [file mededu-v12-e95578-s001.docx]

# Multimedia Appendix 1

Complete LLM Scoring Prompt and Iterative Calibration Procedure

## 1. Overview

This appendix provides the complete scoring prompt used by DeepSeek-V3 to generate automated performance scores in the virtual standardized patient (VSP) platform. The prompt comprises three tasks: (1) checklist-based information gathering scoring (0–50 points), (2) criterion-based communication performance scoring (0–50 points), and (3) summation to produce the final total score (0–100 points). The prompt was iteratively calibrated using 45 independent historical examination records from the 2024 academic year and was frozen before deployment to the study cohort (2024–2025 academic year).

## 2. Patient Simulation Prompt (Doubao-1.5)

The virtual patient dialogue was powered by Doubao-1.5 (ByteDance) with the following system prompt:

Role
You are a professional standardized patient, responsible for simulating a real patient to interact with the user in a Q&A format, in order to assess the user's medical interviewing skills.

Skills
Skill 1: Simulate a Patient Responding to the User's Questions
The patient you are portraying feels confused and anxious about their condition. You must strictly cooperate with the user's questioning.
Answer strictly according to the case information. Do not fabricate any details. If the user asks about information not mentioned in the case, assume it does not exist. For questions involving time or similar details, you may provide a reasonable answer. Do not say "it's not mentioned in the case." When the user asks about general information, do not introduce yourself. Only answer what is asked. For open-ended questions such as "What's wrong?" or "What problem do you have?", respond briefly with only one main symptom, keeping the answer within 20 words.
For specific questions, answer exactly what is asked—do not add extra information. When the user broadly asks for accompanying symptoms for the first time, you may provide one positive symptom. If they ask broadly again about accompanying symptoms, ask them to specify which symptom they mean. Never voluntarily mention negative symptoms (symptoms starting with "no"). If the user asks about multiple symptoms at once, answer them one by one. Use natural, conversational language.

Restrictions
Only answer questions related to the selected case. Do not involve unrelated content.

## 3. Scoring Prompt (DeepSeek-V3)

The following prompt was sent to DeepSeek-V3 (temperature = 0.0) for each student encounter transcript. The model received the full doctor–patient dialogue transcript along with this system prompt:

Role
You are a medical examiner with strong literary expertise. Your tasks are as follows:

Task 1
Based on the provided doctor–patient dialogue transcript, determine one by one whether each specified key piece of information appears in the dialogue. You must examine each item very carefully and must not miss any. As long as the relevant content appears in the dialogue—whether it is asked by the doctor or voluntarily provided by the patient—it should be considered as scored. The use of standard medical terminology is not required. As long as the expressed meaning is consistent with the key information, it counts as correct. For example, "blood in sputum" is equivalent to "hemoptysis." If an approximate time frame appears in the dialogue (such as "worsened a month ago"), it should be considered as fulfilling the requirement for "onset time." There is no need to further ask for a specific date. If a key piece of information cannot be found anywhere in the dialogue, points should be deducted for that item. If it is partially addressed, no points should be deducted. First, list each key piece of information and indicate whether points are deducted. If points are deducted, provide the reason. Then calculate the total deducted points by addition, and subtract the rounded-down total deduction from 50 to obtain the Information Score.

Task 2
Based on the provided doctor–patient dialogue transcript, evaluate the overall consultation performance according to the following criteria.
List each criterion one by one and determine whether points should be deducted. If points are deducted, provide the reason. Add up the total deducted points, and subtract this total from 50 to obtain the Comprehensive Performance Score.
Criteria for Evaluating Consultation Performance:
Clinical reasoning and sequence (excluding repeated questions) – 8 points
Appropriateness of question types – 6 points
Summarization – 6 points
Necessary transitional language – 5 points
Avoidance of repeated questions – 5 points
Avoidance of medical jargon – 4 points
Necessary praise and encouragement – 3 points
Encouraging the patient to ask questions – 3 points
Humanistic care – 5 points
Emotional reassurance – 5 points

Task 3
Add the Information Score and the Comprehensive Performance Score to obtain the patient's final total score.

## 4. Scoring Rubric Specifications

### 4.1 Information Gathering Checklist Items (0–50 points)

The information gathering score is derived from approximately 40 checklist items spanning the following domains. Each item is assessed as present (scored) or absent/missing (deducted). The checklist items are generated per clinical case to match the specific case content.

**Chief complaint characterization:** Onset timing, duration, quality/character, severity, aggravating factors, relieving factors, prior treatment or consultation for this complaint.

**Systematic review of associated symptoms:** Key positive associated symptoms relevant to the chief complaint (e.g., for cough: sputum production, hemoptysis, fever, chest pain, dyspnea). Key negative symptoms used for differential diagnosis.

**Past medical history:** Relevant chronic conditions, prior hospitalizations, surgical history.

**Medication history:** Current medications, recent medication changes, medication allergies or adverse reactions.

**Allergy history:** Drug allergies, food allergies, environmental allergies.

**Family history:** Relevant familial diseases, genetic conditions.

**Social history:** Occupation, smoking history, alcohol consumption, living environment, recent travel or exposure history relevant to the presenting complaint.

### 4.2 Communication Performance Criteria (0–50 points)

**1. Clinical reasoning and sequence (8 points):** Evaluates whether the interview follows a logical clinical reasoning pathway. Questions should be organized in a clinically meaningful sequence (e.g., chief complaint → history of present illness → systematic review → past history). Excludes questions that are repeated (repetition is penalized separately).

**2. Appropriateness of question types (6 points):** Assesses the balance and appropriateness of question formats: appropriate use of open-ended questions at the beginning of new topics, judicious use of closed questions for specific details, and avoidance of leading or suggestive questions that may bias the patient's response.

**3. Summarization (6 points):** Evaluates whether the interviewer periodically summarizes gathered information to verify accuracy with the patient, demonstrates active listening through accurate paraphrasing, and provides a final summary at the end of the history-taking.

**4. Necessary transitional language (5 points):** Assesses the use of appropriate transitional phrases when moving between interview sections (e.g., from history of present illness to past medical history), helping the patient follow the interview structure.

**5. Avoidance of repeated questions (5 points):** Penalizes unnecessary repetition of questions already answered by the patient. Legitimate clarification ("Let me make sure I understood correctly...") is not penalized.

**6. Avoidance of medical jargon (4 points):** Assesses whether the interviewer uses patient-appropriate language and explains medical terms when their use is unavoidable. Technical terms should be accompanied by plain-language explanations.

**7. Necessary praise and encouragement (3 points):** Evaluates whether the interviewer provides appropriate positive reinforcement when the patient provides detailed or helpful information (e.g., "Thank you for describing that so clearly").

**8. Encouraging the patient to ask questions (3 points):** Assesses whether the interviewer actively invites the patient to raise questions, express concerns, or provide additional information not specifically solicited.

**9. Humanistic care (5 points):** Evaluates demonstration of respect for patient dignity, cultural sensitivity, acknowledgment of the patient's experience of illness, and maintenance of a professional, caring demeanor throughout the encounter.

**10. Emotional reassurance (5 points):** Assesses whether the interviewer acknowledges and responds appropriately to the patient's expressed emotional state (anxiety, fear, frustration), provides context-appropriate reassurance, and avoids dismissive or minimizing language.

## 5. Iterative Calibration Procedure

The scoring prompt was calibrated using 45 historical history-taking examination records from the 2024 academic year. These records were drawn from a cohort entirely independent of the 92 students analyzed in this study (2025 academic year), with zero overlap in students, clinical cases, or raters.

**Stage 1: Initial Prompt Drafting:** An initial version of the scoring prompt was drafted based on the existing standardized scoring rubric used in the diagnostics course. The rubric specifications (50/50 weighting, checklist domains, communication criteria) were operationalized into natural-language instructions for the LLM.

**Stage 2: Pilot Testing (n = 15):** The initial prompt was applied to 15 randomly selected historical records. For each record, AI-generated scores (total, information gathering, communication) were compared with the original faculty-assigned scores. Item-level comparisons were conducted to identify specific information checklist items and communication criteria where AI and faculty systematically diverged.

**Stage 3: Discrepancy Analysis:** Systematic scoring discrepancies were identified and categorized: (a) AI over-penalizing minor omissions in information gathering (e.g., deducting points for partially addressed items); (b) AI under-weighting organizational quality and transitional language in communication scoring; (c) AI being insensitive to implicit empathy expressions (acknowledging patient concerns without explicit reassurance phrases).

**Stage 4: Iterative Prompt Refinement:** The prompt was iteratively refined to address each identified discrepancy. For each refinement cycle, the updated prompt was tested on the same 15 records to verify that the targeted discrepancy was reduced without introducing new scoring errors. Specific refinements included: (a) adding the "partially addressed = no deduction" rule for information checklist items; (b) increasing the weight of transitional language and logical sequence in the communication scoring description; (c) adding explicit instruction to recognize implicit empathy expressions.

**Stage 5: Validation (n = 30):** The refined prompt was validated on the held-out 30 historical records to confirm improved overall alignment and to check for overfitting to the calibration subset. Alignment metrics (Spearman ρ, MAE) improved between the initial draft and the final prompt on the held-out set. The final prompt was frozen and deployed without further modification for the duration of data collection.

## 6. Model Configuration

**Scoring model:** DeepSeek-V3 (DeepSeek AI), accessed via API

**Temperature:** 0.0 (deterministic output for scoring)

**Patient dialogue model:** Doubao-1.5 (ByteDance)

**Automatic speech recognition:** paraformer-realtime-v2 (Alibaba)

**Text-to-speech:** CosyVoice-v2 (Alibaba)

**Deployment period:** 2025 academic year

**Prompt status:** Frozen before study data collection; no modifications during study
