# Supplementary material for: Evaluating Large Language Model–Based Automated Scoring in a Voice-Based Virtual Standardized Patient Platform for Medical Students: Cross-Sectional Agreement Study
Source: JMIR Med Educ. 2026 Sep 24;12:e95578. doi: 10.2196/95578 (PMC13606323; doi:10.2196/95578)
Supplement: Supplementary file 2 [file mededu-v12-e95578-s002.docx]

# Multimedia Appendix 2

Detailed Statistical Model Specifications

## 1. Software and Packages

All analyses were conducted in R version 4.3.2 (R Foundation for Statistical Computing, Vienna, Austria). The following packages were used: lme4 (v1.1-35.1) for linear mixed-effects models; lmerTest (v3.1-3) for Satterthwaite degrees of freedom and P-values; irr (v0.84.1) for intraclass correlation coefficients; psych (v2.3.9) for descriptive statistics; ggplot2 (v3.4.4) for visualization; and robustlmm (v3.1-1) for robust mixed-effects estimation.

## 2. Primary Mixed-Effects Model

The primary analysis assessed the association between AI-generated and faculty-assigned total scores using the following linear mixed-effects model:

Human_total_ij = β₀ + β₁ × AI_total_ij + β₂ × Case2_ij + β₃ × Case3_ij + u_j + ε_ij

where i indexes the student (i = 1, …, 92), j indexes the rater (j = 1, …, 10), Human_total_ij is the faculty-assigned total score, AI_total_ij is the AI-generated total score, Case2_ij and Case3_ij are indicator variables for Cases 2 and 3 (Case 1 as reference), u_j ∼ N(0, σ²_u) is the random intercept for rater j, and ε_ij ∼ N(0, σ²_e) is the residual error. The random intercept u_j captures between-rater variability in average scoring severity/leniency. Fixed effects were estimated using restricted maximum likelihood (REML).

## 3. Variance Partition Coefficient (VPC)

The variance partition coefficient was calculated as:

VPC = σ²_u / (σ²_u + σ²_e)

where σ²_u is the between-rater variance component and σ²_e is the residual (within-rater, between-student) variance component. VPC represents the proportion of unexplained variance in human scores attributable to between-rater differences after adjusting for AI score and case. A VPC of 0.37 indicates that 37% of residual variance is at the rater level.

## 4. Model Comparison

The mixed-effects model (with random rater intercepts) was compared against an ordinary least squares model (without random effects) using Akaike's Information Criterion (AIC). The mixed-effects model showed improved fit (ΔAIC = 19.10), supporting the inclusion of rater-level random effects.

## 5. Intraclass Correlation Coefficient

Absolute agreement between AI and human total scores was quantified using ICC(2,1): a two-way random-effects model, single-measure, absolute agreement definition. This ICC variant treats both AI and human raters as random effects drawn from a population of possible raters and assesses the absolute (rather than consistency) agreement of single measurements. The ICC(2,1) was computed using the irr package with the formula:

ICC(2,1) = (MS_S - MS_E) / (MS_S + (k-1) × MS_E + k × (MS_R - MS_E) / n)

where MS_S is the between-subject mean square, MS_R is the between-rater mean square, MS_E is the residual mean square, k = 2 (number of measurements per subject: AI and human), and n = 92 (number of subjects). Confidence intervals were computed using the F-distribution method (Shrout & Fleiss, 1979).

## 6. Mixed-Effects Bland–Altman Analysis

The Bland–Altman analysis accounted for rater clustering by fitting a linear mixed-effects model to the paired differences:

(Human_total_ij − AI_total_ij) = β₀ + β_mean × Mean_ij + u_j + ε_ij

where Mean_ij = (Human_total_ij + AI_total_ij) / 2. The intercept β₀ estimates the overall mean bias. The coefficient β_mean tests for proportional bias (i.e., whether the difference depends on the magnitude of the measurement). The 95% limits of agreement were calculated as:

95% LoA = β₀ ± 1.96 × σ_e

where σ_e is the residual standard deviation from the mixed-effects model (conditional on rater). This approach yields narrower, more appropriate limits of agreement than the standard Bland–Altman method, as it accounts for the clustered data structure.

## 7. Random-Slope Model

We explored whether the AI–human association varied across raters by fitting a random-slope model:

Human_total_ij = β₀ + β₁ × AI_total_ij + β₂ × Case2_ij + β₃ × Case3_ij + u_0j + u_1j × AI_total_ij + ε_ij

This model yielded a singular fit (estimated correlation between random intercept and random slope = ±1.00, random-slope variance ≈ 0), indicating that there was insufficient information to estimate meaningful between-rater variability in the AI–human slope. This suggests that rater heterogeneity primarily operates through intercept shifts (severity/leniency differences) rather than through differential AI–human associations across raters.

## 8. Subscale Models

All primary models were repeated separately for the information gathering and communication subscales (each 0–50 points). The model specifications were identical to the total score model, substituting the respective subscale scores as dependent and independent variables.

## 9. Sensitivity Analyses

### 9.1 Robust Mixed-Effects Estimation

To evaluate the influence of potential outliers, the primary model was refit using robust mixed-effects estimation (robustlmm package, rlmer function with smooth Huber Ψ-function). This approach down-weights observations with large residuals, providing estimates that are less sensitive to extreme values. The AI coefficient from the robust model (β_robust = 0.35, P < .001) was essentially unchanged from the primary REML estimate (β = 0.37).

### 9.2 Model Without Case Adjustment

The primary model was refit without case adjustment: Human_total ~ AI_total + (1|Rater). The AI coefficient (β = 0.36, P < .001) was essentially unchanged from the case-adjusted model, consistent with the non-significant case effects in the primary analysis.

### 9.3 Lower-Quartile Sensitivity Analysis

To assess the impact of ceiling effects on agreement estimates, we conducted a sensitivity analysis restricted to students in the lowest quartile of the pooled (AI + human) score distribution. The 25th percentile of the pooled distribution was 90.0 points. A total of 22 students fell at or below this threshold. All agreement metrics (LMM, ICC, Spearman ρ, Bland–Altman) were recomputed within this subgroup. Results are reported in Appendix 3, Table A3.

### 9.4 Range-Restriction Correction

We applied Thorndike's Case 2 correction for direct range restriction to estimate the ICC in an unrestricted population:

ICC_unrestricted = ICC_restricted × (SD_unrestricted / SD_restricted) / sqrt(1 − ICC²_restricted + ICC²_restricted × (SD²_unrestricted / SD²_restricted))

The reference unrestricted SD (SD_unrestricted ≈ 8.5) was drawn from published multi-institution OSCE total score data (Brannick et al., 2011 [10]). The study SD (SD_restricted ≈ 5.3 for total scores) was used. Applying this correction yielded a corrected ICC of approximately 0.72. We emphasize that this extrapolation relies on the assumption that the reference population SD is appropriate and that the relationship between AI and human scores is linear across the full range; these assumptions are unverified and the corrected estimate should be treated as exploratory only.

## 10. Descriptive Statistics

Given the bounded (0–100) nature of the scores and the observed right-skew with ceiling tendencies, results were summarized using median and interquartile range (IQR) rather than mean and standard deviation. All tests were two-sided with α = .05. Bootstrap confidence intervals for Spearman's ρ were computed using 10,000 resamples with bias-corrected and accelerated (BCa) method.
