# Supplementary material for: Evaluating Large Language Model–Based Automated Scoring in a Voice-Based Virtual Standardized Patient Platform for Medical Students: Cross-Sectional Agreement Study
Source: JMIR Med Educ. 2026 Sep 24;12:e95578. doi: 10.2196/95578 (PMC13606323; doi:10.2196/95578)
Supplement: Supplementary file 3 [file mededu-v12-e95578-s003.docx]

# Multimedia Appendix 3

Score Distribution Histograms and Student Feedback Survey Results

## Part A: Score Distribution Histograms

Figure S1 displays the distributions of AI-generated and human-assessed total scores (0–100). Both distributions show right-skewed patterns with ceiling effects, consistent with the high-performing study cohort. The dashed red line indicates the 95-point threshold used for ceiling effect quantification (18% of both AI and human scores above this threshold). The solid blue line indicates the median (AI: 93.0; human: 94.0).

Figure S2 displays the corresponding histograms for the information gathering (0–50) and communication (0–50) subscales. Subscale distributions are compressed but do not show the same degree of ceiling saturation as total scores.

## Part B: Student Feedback Survey Results

A post-encounter online survey was administered to all 92 study participants. A total of 63 students responded (68% response rate). The survey assessed usage patterns, system quality perceptions, perceived learning benefits, comparison with human standardized patients, overall satisfaction, and behavioral intentions. All items were rated on 1–5 Likert scales unless otherwise noted.

### B.1 Respondent Demographics and Usage

Total respondents: 63 out of 92 (68%)

VSP usage frequency: 1 session: 9 (14%); 2 sessions: 35 (56%); 3 sessions: 19 (30%). Students with ≥2 sessions: 54 (86%).

Primary purpose (multiple choice): A) Self-directed practice: 29 (46%); B) Exam preparation: 51 (81%); C) Assigned task: 28 (44%); D) Curiosity about new technology: 21 (33%).

### B.2 System Quality Perceptions (1–5 scale)

| **Item** | **n** | **Mean** | **Median** | **Rating ≥4 (%)** | **Rating ≤2 (%)** |
| --- | --- | --- | --- | --- | --- |
| Voice recognition accuracy | 63 | 3.2 | 3.0 | 48% | 22% |
| Response relevance | 63 | 4.0 | 4.0 | 84% | 8% |
| Response realism (medical/logical plausibility) | 63 | 2.8 | 3.0 | 29% | 38% |
| Voice naturalness | 63 | 2.9 | 3.0 | 30% | 35% |
| Dialogue fluency | 63 | 2.8 | 3.0 | 35% | 36% |
| System ease of use | 63 | 3.8 | 4.0 | 75% | 13% |

### B.3 Perceived Learning Benefits (1–5 scale)

| **Item** | **n** | **Mean** | **Median** | **Rating ≥4 (%)** | **Rating ≤2 (%)** |
| --- | --- | --- | --- | --- | --- |
| Familiarity with interview | 63 | 3.9 | 4.0 | 71% | 8% |
| Skill improvement (questioning and communication techniques) | 63 | 3.4 | 3.0 | 46% | 21% |
| Knowledge consolidation (disease understanding and memory) | 63 | 3.4 | 3.0 | 49% | 19% |
| Confidence building | 63 | 3.3 | 3.0 | 44% | 19% |
| Pre-exam stress reduction | 63 | 3.3 | 3.0 | 49% | 24% |
| Self-reflection (via transcript review) | 63 | 3.9 | 4.0 | 75% | 11% |

### B.4 Comparison with Human Standardized Patients (1–5 scale)

| **Item** | **n** | **Mean** | **Median** | **Rating ≥4 (%)** | **Rating ≤2 (%)** |
| --- | --- | --- | --- | --- | --- |
| Relaxation (less fear of making mistakes vs. human SP) | 63 | 3.2 | 4.0 | 54% | 30% |
| Feedback value (AI written feedback vs. human SP face-to-face feedback) | 63 | 2.4 | 2.0 | 21% | 52% |
| Realism and immersion (vs. human SP encounters) | 63 | 1.8 | 2.0 | 5% | 86% |
| Convenience (anytime/anywhere practice) | 63 | 4.1 | 4.0 | 81% | 11% |

### B.5 Overall Satisfaction and Behavioral Intentions (1–10 scale)

| **Item** | **n** | **Mean** | **Median** | **Min** | **Max** | **Rating ≥8 (%)** |
| --- | --- | --- | --- | --- | --- | --- |
| Overall satisfaction with VSP experience | 63 | 6.4 | 7.0 | 1 | 10 | 32% |
| Willingness to continue using VSP | 63 | 6.0 | 6.0 | 1 | 10 | 33% |
| Willingness to recommend VSP to peers | 63 | 5.8 | 6.0 | 1 | 10 | 30% |

### B.6 Qualitative Feedback Summary

Open-ended responses (Questions 9–11) were reviewed and thematically organized. Representative themes are summarized below.

Positive themes:

- Convenience and accessibility: Students valued the ability to practice anytime and anywhere, with self-paced repetition without time pressure. "Can practice anytime, psychological pressure is lower."
- Detailed written feedback: Students appreciated being able to review the full transcript and see itemized feedback. "Feedback is meticulous. Can discover my own deficiencies by reviewing the transcript."
- Examination preparation: The VSP was seen as useful for familiarizing with the interview before formal examinations. "More practice opportunities, feeling more confident."
- Transcript review: The voice-to-text recording feature was valued for post-hoc self-reflection. "Can view the consultation process in text form."

Areas for improvement:

- ASR accuracy: Voice recognition errors were the most frequently reported issue, particularly for medical terminology, rapid speech, and premature speech cutoff. "Often couldn't hear clearly," "Voice recognition errors occurred frequently."
- Lack of conversational naturalness: Students reported that the AI patient responses felt mechanical, repetitive, and lacked the nuance of real patient interactions. "AI's way of answering is quite different from real people." "Overall performance inferior to real SP, with a strong 'artificial' feeling."
- Rigid scoring: Checklist-based scoring was perceived as overly rigid, penalizing semantically equivalent expressions. "Scoring standards too rigid — need specific keywords or points are deducted."
- Low perceived realism: Immersion and realism were the lowest-rated dimensions. "The sense of immersion is lacking." "Answers sometimes obviously don't match common sense."
- Technical issues: Login difficulties, VPN requirements, and latency were noted as barriers. "Login system had too many bugs." "Delay was high."

These qualitative findings are consistent with the quantitative ratings: convenience and self-reflection were the highest-rated benefits, while realism/immersion and feedback value (relative to human SP feedback) were the lowest-rated dimensions.
