# Supplementary material for: Evaluating Large Language Model–Based Automated Scoring in a Voice-Based Virtual Standardized Patient Platform for Medical Students: Cross-Sectional Agreement Study
Source: JMIR Med Educ. 2026 Sep 24;12:e95578. doi: 10.2196/95578 (PMC13606323; doi:10.2196/95578)
Supplement: Supplementary file 4 [file mededu-v12-e95578-s004.docx]

# Multimedia Appendix 4

Supplementary Tables: Rater-Stratified Agreement Metrics, Sensitivity Analyses, and Lower-Quartile Analysis

## Table A1. Rater-Stratified AI–Human Agreement Metrics (Total Score)

| **Rater** | **n** | **AI Median** | **Human Median** | **Bias (H−A)** | **Spearman ρ** | **MAE** | **ICC(2,1)*** |
| --- | --- | --- | --- | --- | --- | --- | --- |
| 1 | 9 | 94.0 | 95.0 | 1.33 | 0.49 | 2.44 | 0.69 |
| 10 | 10 | 89.0 | 92.0 | 3.20 | 0.63 | 3.40 | 0.86 |
| 2 | 9 | 94.0 | 94.0 | -1.39 | 0.50 | 2.17 | 0.87 |
| 3 | 9 | 93.0 | 95.0 | 2.67 | 0.26 | 3.11 | 0.57 |
| 4 | 9 | 93.0 | 91.0 | -1.22 | 0.65 | 2.56 | 0.86 |
| 5 | 8 | 93.0 | 94.5 | 3.25 | 0.21 | 5.00 | 0.75 |
| 6 | 10 | 93.0 | 94.0 | 2.20 | 0.92 | 2.80 | 0.80 |
| 7 | 10 | 91.0 | 87.2 | -2.05 | 0.79 | 4.15 | 0.88 |
| 8 | 10 | 92.5 | 91.2 | -0.15 | 0.76 | 3.05 | 0.93 |
| 9 | 8 | 93.0 | 95.0 | 1.75 | 0.38 | 2.50 | 0.78 |

* ICC(2,1) computed per rater with n = 8–10 observations. These per-rater estimates have wide confidence intervals given the very small sample sizes and should be interpreted descriptively only. Negative ICC estimates are reported as observed but are not meaningful.

## Table A2. Lower-Quartile Sensitivity Analysis (Total Score)

| **Metric** | **Full Sample (n = 92)** | **Lower Quartile (n = 22)** | **Upper 75% (n = 70)** | **Change** |  |
| --- | --- | --- | --- | --- | --- |
| Sample size | 92 | 22 | 70 | — |  |
| AI total, mean (SD) | 91.9 (4.6) | 86.1 (4.6) | 93.7 (2.8) | — |  |
| Human total, mean (SD) | 92.8 (3.4) | 88.4 (2.5) | 94.2 (2.2) | — |  |
| Bias (Human − AI) | 0.92 | 2.23 | 0.51 | Attenuated in low quartile |  |
| MAE | 3.11 | 4.64 | 2.64 | Larger at low end |  |
| Spearman ρ | 0.50 | -0.14 | 0.13 | Attenuated |  |
| ICC(2,1) | 0.51 | 0.38 | 0.55 | Attenuated |  |

Note: The lower quartile was defined as students with a mean (AI + Human)/2 total score ≤ 90.0 (25th percentile of the pooled distribution). Wider confidence intervals in the lower quartile reflect reduced sample size (n = 22). The full-set ICC(2,1) = 0.51 is attenuated by ceiling effects; the lower-quartile ICC(2,1) = 0.38 is further attenuated by range restriction within the already-restricted lower segment.

Bland–Altman metrics (full sample): mean bias = 1.26 points, 95% limits of agreement = −4.95 to 7.48 (width = 12.43 points), proportional bias β_proportional bias = −0.55 (P<.001).

## Table A3. Sensitivity Analyses: Robust Mixed Model and Without Case Adjustment

| **Model Specification** | **β (AI→Human)** | **95% CI** | **P** |
| --- | --- | --- | --- |
| Primary: AI_total + Case + (1\|Rater) | 0.37 | (0.26, 0.48) | < .001 |
| Robust mixed model | 0.35 | (0.24, 0.46) | < .001 |
| Without case: AI_total + (1\|Rater) | 0.36 | (0.25, 0.47) | < .001 |

Note: All models used restricted maximum likelihood (REML) estimation with random rater intercepts. The robust model used smooth Huber Ψ-function (rlmer, robustlmm package). AI coefficients were essentially unchanged across specifications.
